# Regulation of food intake by astrocytes in the brainstem dorsal vagal complex

**DOI:** 10.1002/glia.23774

**Published:** 2019-12-27

**Authors:** Alastair J. MacDonald, Fiona E. Holmes, Craig Beall, Anthony E. Pickering, Kate L. J. Ellacott

**Affiliations:** ^1^ Institute of Biomedical and Clinical Sciences University of Exeter Medical School Exeter UK; ^2^ School of Physiology, Pharmacology and Neuroscience University of Bristol, Biomedical Sciences Building, University Walk Bristol UK; ^3^ Anaesthesia, Pain and Critical Care Sciences, Translational Health Sciences Bristol Medical School, University of Bristol Bristol UK

**Keywords:** astrocyte, chemogenetic, feeding, metabolism, nucleus of the solitary tract

## Abstract

A role for glial cells in brain circuits controlling feeding has begun to be identified with hypothalamic astrocyte signaling implicated in regulating energy homeostasis. The nucleus of the solitary tract (NTS), within the brainstem dorsal vagal complex (DVC), integrates vagal afferent information from the viscera and plays a role in regulating food intake. We hypothesized that astrocytes in this nucleus respond to, and influence, food intake. Mice fed high‐fat chow for 12 hr during the dark phase showed NTS astrocyte activation, reflected in an increase in the number (65%) and morphological complexity of glial‐fibrillary acidic protein (GFAP)‐immunoreactive cells adjacent to the area postrema (AP), compared to control chow fed mice. To measure the impact of astrocyte activation on food intake, we delivered designer receptors exclusively activated by designer drugs (DREADDs) to DVC astrocytes (encompassing NTS, AP, and dorsal motor nucleus of the vagus) using an adeno‐associated viral (AAV) vector (AAV‐GFAP‐hM3Dq_mCherry). Chemogenetic activation with clozapine‐*N*‐oxide (0.3 mg/kg) produced in greater morphological complexity in astrocytes and reduced dark‐phase feeding by 84% at 4 hr postinjection compared with vehicle treatment. hM3Dq‐activation of DVC astrocytes also reduced refeeding after an overnight fast (71% lower, 4 hr postinjection) when compared to AAV‐GFAP‐mCherry expressing control mice. DREADD‐mediated astrocyte activation did not impact locomotion. hM3Dq activation of DVC astrocytes induced c‐FOS in neighboring neuronal feeding circuits (including in the parabrachial nucleus). This indicates that NTS astrocytes respond to acute nutritional excess, are involved in the integration of peripheral satiety signals, and can reduce food intake when activated.

## MAIN POINTS

- High‐fat feeding in mice induces morphological changes in brainstem astrocytes.
- Chemogenetic activation of astrocytes in the dorsal vagal complex reduces feeding.
- Glial activation recruits local neural circuits known to be involved in food intake.

Chemogenetic activation of astrocytes in the mouse dorsal vagal complex reduces food intake and activates local neuronal circuits

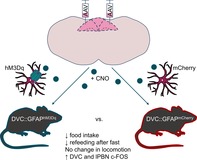

## 1 INTRODUCTION

Food intake is controlled by the coordinated action of numerous brain regions but a complete understanding remains elusive (Andermann & Lowell, 2017). The brainstem dorsal vagal complex (DVC) is the first site for integration of visceral synaptic and hormonal cues that act to inhibit food intake (Grill & Hayes, 2012). The DVC consists of three nuclei: the nucleus of the solitary tract (NTS), area postrema (AP), and dorsal motor nucleus of the vagus. Neurons of the sensory branch of the vagus nerve make glutamatergic synapses onto NTS neurons to relay information from the periphery, including the stomach and upper intestine (Doyle & Andresen, 2001; Williams et al., 2016). Targeted chemogenetic activation of appetite‐responsive NTS neuronal populations causes short term decreases in food intake (Cerritelli, Hirschberg, Hill, Balthasar, & Pickering, 2016; D'Agostino et al., 2016; Gaykema et al., 2017; Roman, Derkach, & Palmiter, 2016; Zhan et al., 2013).

Astrocytes provide metabolic and structural support to neurons and play an active role in modulating neurotransmission (Verkhratsky & Nedergaard, 2018). Selective manipulation of astrocyte populations, including chemogenetic modulation, has revealed diverse roles of these cells in many physiological and behavioral responses including autonomic control (Agulhon et al., 2013; Sciolino et al., 2016), addiction (Bull et al., 2014; Scofield et al., 2015), fear conditioning (Martin‐Fernandez et al., 2017), and hippocampus‐dependent learning (Adamsky et al., 2018). Astrocytes within the hypothalamic arcuate nucleus (ARC) are regulated by both positive and negative energy balance as shown by increased expression of glial‐fibrillary acidic protein (GFAP), morphological plasticity (increased branching and complexity of astrocyte processes) and altered expression of astrocyte‐specific neurotransmitter and glucose transporters (Buckman et al., 2015; Chen et al., 2016; Fuente‐Martín et al., 2012; Zhang, Reichel, Han, Zuniga‐Hertz, & Cai, 2017). Optogenetic and chemogenetic activation of ARC astrocytes alters food intake by manipulating the firing of hunger‐driving agouti‐related peptide (AgRP) neurons (Chen et al., 2016; Sweeney, Qi, Xu, & Yang, 2016; Yang, Qi, & Yang, 2015). Hypothalamic astrocytes also express receptors for hormones that influence satiety and hunger, suggesting that this population of cells is directly sensitive to energy state (Cheunsuang & Morris, 2005; Fuente‐Martín et al., 2016; García‐Cáceres et al., 2016; Kim et al., 2014).

Given the similarities between the NTS and the ARC, namely that both nuclei contain neurons that exert powerful effects on feeding behavior and are adjacent to circumventricular organs, we hypothesized that NTS astrocytes may be involved in mediating satiety. In support of this hypothesis NTS astrocytes sense synaptic input from the vagus nerve, which drives intracellular Ca^2+^ increases in ex vivo brain slices (McDougal, Hermann, & Rogers, 2011). Furthermore, NTS astrocytes are implicated in the appetite suppressing effects of the glucagon‐like peptide 1 (GLP‐1) receptor agonist exendin‐4 (Reiner et al., 2016). However, what remains unknown is whether NTS astrocytes respond to physiological perturbations in energy homeostasis. Furthermore, there has not been a causal demonstration of the impact of NTS astrocyte activation on physiological feeding behavior. To address these key questions, we used a 12‐hr high‐fat feeding paradigm to trigger a feeding binge and examined morphological changes in NTS astrocytes. We then targeted the expression of excitatory designer receptors exclusively activated by designer drugs (DREADDs) to DVC astrocytes to allow specific chemogenetic activation of these cells and determined the impact on feeding behavior in mice.

## 2 METHODS

### 2.1 Mice

All animal studies were conducted in accordance with the UK Animals in Scientific Procedures Act 1986 (ASPA) and study plans were approved by the institutional Animal Welfare and Ethical Review Body at the University of Bristol and/or Exeter. Adult male C57BL6/J mice (Charles River, UK) were used for all experiments. Unless stated otherwise, mice were group housed on a 12:12 light–dark cycle at 22 ± 2°C, with unlimited access to standard laboratory rodent diet (EURodent diet [5LF2], LabDiet, UK) and water.

### 2.2 Dark‐phase high‐fat feeding studies and histology

**Figure 1:**
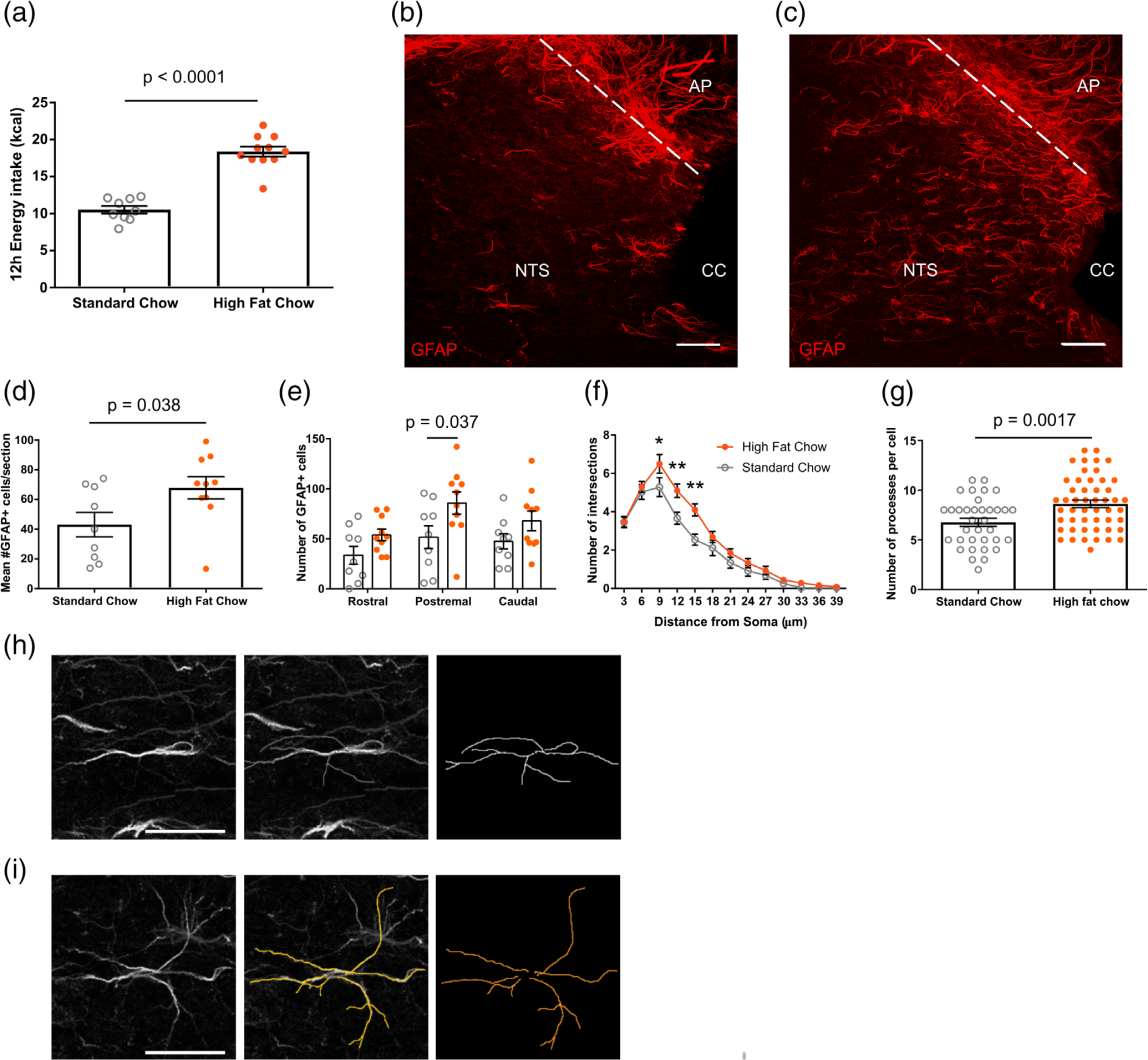
High‐fat chow intake increased the number and morphological complexity of astrocytes in the nucleus of the solitary tract (NTS). (a) Dark‐phase energy intake of standard and high‐fat chow fed mice (10.53 ± 0.51 vs. 18.37 ± 0.67 kcal, *n* = 9–10 mice/group, *p* < .0001, unpaired *t* test). (b,c) Representative maximum projection confocal image of glial‐fibrillary acidic protein (GFAP) immunostaining from a standard (b) and high‐fat (c) chow fed mouse, scale bar = 50 μm. (d) Mean number of GFAP positive cells from tissue sections of NTS from standard and high‐fat chow fed mice (43.09 ± 8.19 vs. 67.9 ± 7.47 cells, *n* = 9–10 mice/group, *p* = .038, unpaired *t* test). (e) Number of GFAP positive cells within anatomical subdivisions of NTS from standard (open gray circle) and high‐fat (closed orange circle) chow fed mice (*n* = 9–10 mice/group, two‐way analysis of variance [ANOVA], food, *p* = .0018, *F*_(1,51)_ = 10.81; rostrocaudal position, *p* = .034, *F*_(2,51)_ = 3.63; interaction, *p* = .7, *F*_(2,51)_ = 0.36; Sidak's post hoc test). (f) Mean Sholl profile of postremal NTS astrocytes of standard and high‐fat chow fed mice (*n* = 35–50 cells from 4–5 mice/group, two‐way ANOVA, food, *p* < .0001, *F*_(1,1,079)_ = 23.24; distance from soma, *p* < .0001, *F*_(12,1,079)_ = 108.3; interaction, *p* = .04, *F*_(12,1,079)_ = 1.83; Sidak's post hoc test). (g) Number of processes of individual postremal NTS astrocytes from standard and high fat chow fed mice (6.77 ± 0.41 vs. 8.62 ± 0.38 processes, *n* = 35–50 cells from 4–5 mice/group, *p* = .0017, unpaired *t* test). (h,i) Representative image and trace of a GFAP+ astrocyte from a standard (h) and high‐fat (i) chow fed mouse, scale bar = 25 μm. *p < .05, **p < .01. AP, area postrema; cc, central canal; NTS, nucleus of the solitary tract. Data are expressed as mean ± *SE* of the mean [Color figure can be viewed at http://wileyonlinelibrary.com]

Two independent cohorts of mice (aged 16 weeks) were individually housed for 4–5 days. At lights‐off on the test day, in the experimental group, standard diet was substituted for high‐fat chow (D12492, TestDiet) while control mice remained on standard chow (EURodent diet). Then, 12–14 hr later mice were euthanized (with sodium pentobarbital) and transcardially perfused with heparinized 0.9% saline then 4% paraformaldehyde. Brains were postfixed in 4% paraformaldehyde for 4–6 hr before being transferred to 30% sucrose in phosphate buffered saline (PBS). Then, 30 μm coronal sections of the brainstem were taken with a freezing sledge microtome (Bright instruments, UK) in four series. One series was stained with mouse anti‐GFAP (MAB360; Millipore, UK) followed by donkey anti‐mouse Alexa Fluor 568 (A10037; Invitrogen, UK) which allowed for GFAP‐immunoreactivity to be visualized (Buckman, Thompson, Moreno, & Ellacott, 2013). Omission of the primary antibody was used to confirm that the immunoreactivity seen was not due to nonspecific binding of the secondary antibody to the tissue (Supplementary Figure S1a). Stained sections were mounted onto glass slides and coverslipped with fluoroshield mounting medium with DAPI (ab104139; Abcam, UK). Images for cell counting were acquired at 10× magnification on a fluorescence microscope (DM 4000; Leica, Germany). The NTS was subdivided (Figure 1e) as follows: rostral = Bregma −7.08 to −7.2 mm, postremal = Bregma −7.32 to −7.64 mm, caudal = Bregma −7.76 to −8 mm (Paxinos & Franklin, 2001). Images for morphological analysis were acquired at 20× magnification on a confocal microscope (DMi8; Leica). Images were analyzed in FIJI (Schindelin et al., 2012) (National Institutes of Health) using the cell counter, simple neurite tracer, and Sholl analysis plugins (Ferriera et al., 2014). For tracing, five cells per z‐stack from two z‐stacks per mouse were randomly selected for tracing and morphological analysis. The investigator was blinded to the diet of the mice during immunohistochemical staining, image acquisition, and analysis.

### 2.3 NTS viral vector injection

Mice (8–12 weeks) were injected with adeno‐associated viral (AAV) vectors in the NTS as described previously (Cerritelli et al., 2016). The vectors used were AAV5/2‐hGFAP‐hM3Dq_mCherry (v97‐5, titer ≥7.5 × 10^12^ viral genomes/ml; University of Zurich Viral Vector Facility, Switzerland) and AAV5/2‐hGFAP‐mCherry (custom preparation, titer 3.89 × 10^13^ viral genomes/ml; ViGene Biosciences) diluted 1:1 in sterile PBS prior to injection. In brief, mice were deeply anaesthetized with an intraperitoneal (i.p.) injection of ketamine (70 mg/kg) and medetomidine (0.5 mg/kg) and placed in a stereotaxic frame (David Kopf Instruments) with the nose angled down by 20°. An incision from the occiput to the nape of the neck was made, the muscles were parted, and the atlantooccipital membrane incised to expose the surface of the brainstem. Injections were made from a pulled glass pipette attached to an injection system (Neurostar, Germany) mounted on the stereotaxic frame at an angle of 35° to the vertical, tip facing rostral. The pipette was inserted 400 μm lateral to the midline at the level of calamus scriptorius to a depth of 1 mm. Four AAV injections of 180 nl were made bilaterally at angled depths of 1 mm, 750 μm, 500 μm, and 250 μm, respectively, at a rate of 100 nl/min. The pipette was left in place for 1 min after each injection. A second, independent cohort of mice underwent the same procedure but with a single 180 nl viral injection per side at 200 μm lateral to the midline at a depth of 500 μm (Supplementary Figure S5). Mice had atipamezole (1 mg/kg i.p.) and buprenorphine (0.1 mg/kg subcutaneous) for anesthetic reversal and analgesia, respectively, and were transferred to a heated cage to recover. Following surgery mice were individually housed for the duration of the experiment. The investigator was blinded to mouse group allocations for all the subsequent experiments.

### 2.4 Feeding assays

At 4–8 weeks following surgery, mice were acclimatized to experimenter handling and i.p. injections of 0.9% NaCl (saline) daily for 4 days. For dark‐phase feeding studies, mice were given an i.p. injection of saline 15–30 min prior to the beginning of the dark phase. Food intake was manually measured by the investigator (under red‐light illumination where necessary) at 2, 4, 6, 12, and 24 hr after lights‐off. Body weight was measured 8 hr after lights‐off. The following day mice were given an injection of clozapine‐*N*‐oxide (CNO, 0.3 mg/kg i.p., Tocris, UK; diluted in saline) and food intake and body weight were measured at the same time points. For fast‐induced refeeding experiments, food was removed from cages at the beginning of the dark phase. Subsequently, 15–30 min prior to the onset of light‐phase (11.5–11.75 hr later), mice received an injection of CNO (0.3 mg/kg i.p.). After injection, food was returned to cages at the onset of the light phase and intake was measured 1, 2, 4, 6, 8, 12, and 24 hr after lights‐on. Body weight was measured twice: once prior to food being removed and once at 8 hr after the reintroduction of food.

### 2.5 Conditioned place aversion assay

For conditioned place aversion (CPA) testing, an apparatus consisting of two distinct chambers joined by a clear plastic external corridor (see Figure 4b) was used. The left chamber had horizontal black and white stripes on the walls and a perforated floor, and the right chamber had vertical black and white stripes on the walls and a floor with horizontal grating. A counterbalanced paradigm was used to test CPA (see Figure 4a). On the first day, mice were given 20 min of free access to explore the whole apparatus. This session was recorded using a video camera and used to determine initial location preferences. On the second day (Conditioning 1), mice were assigned to receive either CNO (0.3 mg/kg i.p.) or an equivalent volume of saline 15 min prior to being placed in either the left or right chamber for 45 min, with the access to the external corridor and second chamber blocked. On the third day (Conditioning 2), mice received the opposite treatment with their access restricted to the alternate chamber, compared to the second day. On the fourth/final day, mice were given free access to the whole apparatus for 30 min. Again, this session was recorded and used to determine conditioned preference. Recorded sessions were analyzed offline using Ethovision XT (Noldus, Netherlands) tracking software. An independent cohort of C57BL6/J mice (*n* = 7) underwent the same conditioning protocol with the known aversive agent lithium chloride (LiCl, 150 mg/kg i.p. diluted in saline) replacing CNO as the experimental stimulus.

### 2.6 Home cage food seeking

Individually housed mice were injected with CNO (0.3 mg/kg i.p.) 15 min prior to the beginning of the dark phase and their home cage placed under a video camera with food removed from the hopper and two pieces of food placed on the cage floor in the far corner from the nest. The activity was then recorded for 3 hr and food intake at 3 hr was measured. Recorded sessions were analyzed offline using Ethovision XT tracking software with a 6 cm^2^ zone centered around the pellets termed the “food zone.”

### 2.7 Validation of DREADD transduction of astrocytes and evaluation of DREADD‐induced c‐FOS expression

Mice received CNO (0.3 mg/kg i.p.) during the first 4 hr of the light phase and had food removed from the cage. After 2–3 hr, mice underwent transcardial perfusion fixation as described above. Then, 30 μm coronal sections were taken and stained with primary antibodies against GFAP, mCherry, NeuN, and c‐FOS (Table 1) followed by the appropriate secondary antibodies: donkey anti‐mouse Alexa Fluor488, donkey anti‐goat Alexa Fluor594, and donkey anti‐rabbit Alexa Fluor488 (Invitrogen). Omission of primary antibody was used to confirm that the immunoreactivity seen was not due to non‐specific binding of the secondary antibody to the tissue (Supplementary Figure S1b,c). For double immunohistochemistry, mCherry staining was performed first followed by either GFAP or NeuN. Images were acquired on a confocal microscope (DMi8; Leica). For a subset of DVC::GFAP^hM3Dq^ mice (*n* = 4), the number of GFAP or NeuN cells expressing mCherry were counted and calculated as a percentage of total GFAP or NeuN expressing cells. For c‐FOS quantification, images were analyzed in FIJI (Schindelin et al., 2012) (NIH) with the cell counter plugin. Sections containing paraventricular nucleus of the hypothalamus (PVH; Bregma −0.94 mm), lateral parabrachial nucleus (lPBN; Bregma −5.02 mm) and NTS/AP (Bregma −7.56 mm) were analyzed. Morphological analysis of astrocytes from CNO‐treated mice (Supplementary Figure S3) was performed as described above (Section 2.2, histology). The distribution of mCherry transduction of cells (Supplementary Figure S2) was mapped relative to corresponding sections in a reference atlas (Paxinos & Franklin, 2001) and area of transduction quantified using Fiji. The investigator was blinded to the group of the mice during immunohistochemical staining, image acquisition, cell counting, and morphological analysis.

**Table 1:** Antibodies used in the study

Antibody	Dilution	Identifier	Manufacturer
Mouse anti‐GFAP	1:5,000	MAB360	Merck, UK
Rabbit anti‐NeuN	1:2,000	ab177487	Abcam, UK
Goat anti‐mCherry	1:1,000	AB0040‐200	SICGEN, Portugal
Rabbit anti‐c‐FOS	1:2,000	2250S	Cell Signaling Technologies, UK

Abbreviation: GFAP, glial‐fibrillary acidic protein.

### 2.8 Statistical analysis

Data are presented as mean ± standard error (SE) of the mean where appropriate. Data for all experiments were analyzed in Prism 7 (Graph Pad) and the appropriate statistical tests conducted. Graphs were generated in Prism and figures prepared in Inkscape (http://inkscape.org).

## 3 RESULTS

### 3.1 Dark‐phase high‐fat chow feeding induced structural plasticity in NTS astrocytes

In order to examine whether NTS astrocytes respond to changes in energy status, we induced short‐term positive energy balance by allowing experimental mice to exclusively eat a high‐fat chow for 12 hr during the dark phase. Control mice were maintained on standard chow. This paradigm induced a feeding binge in the high‐fat fed mice, with the animals eating 74% more calories when compared with standard chow‐fed mice (Figure 1a). High‐fat fed mice had a greater number of GFAP‐immunoreactive astrocytes within the NTS (58% greater) when compared with standard chow fed controls, with the changes being most pronounced in the postremal NTS (Figure 1b–e). GFAP‐immunoreactive astrocytes in the postremal NTS of high‐fat fed mice had greater morphological complexity, as assessed by Sholl analysis (Ferriera et al., 2014; Sholl, 1953), and a greater number of processes than those of standard chow fed control mice (Figure 1f–i). These findings suggest that NTS astrocytes show dynamic, reactive changes to the acute nutritional excess caused by consumption of a high‐fat diet.

### 3.2 Chemogenetic activation induced structural changes in NTS astrocytes

**Figure 2:**
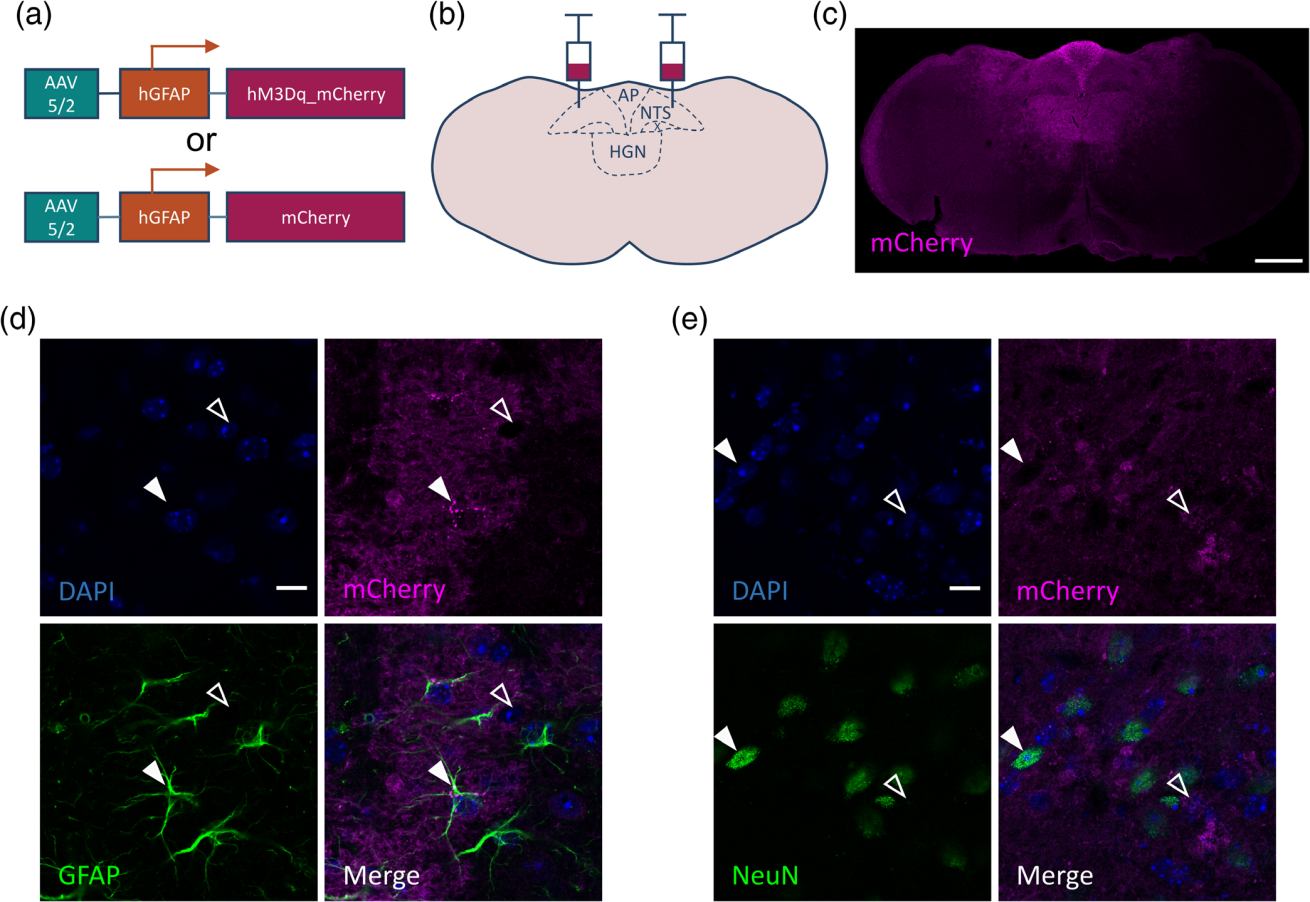
Expression of hM3Dq_mCherry in dorsal vagal complex (DVC) astrocytes. (a) adeno‐associated viral (AAV) vectors containing either hM3Dq_mCherry or mCherry under the hGFAP promoter. (b) Schematic of bilateral injection of the vector into the DVC. (c) Representative image showing mCherry immunofluorescence in a DVC::GFAP^hM3Dq^ mouse, scale bar = 500 μm. (d,e) Immunoreactivity for glial‐fibrillary acidic protein (GFAP) (d) or NeuN (e) and mCherry in a DVC::GFAP^hM3Dq^ mouse. Closed arrow in (d) shows a GFAP‐positive cell, open arrow shows a GFAP‐negative putative neuron while closed arrow in (e) shows a NeuN‐positive neuron, open arrow shows a NeuN‐negative putative astrocyte. Scale bar = 25 μm. 91.5 ± 1.6% of GFAP+ cells had mCherry immunoreactivity (242 of 265 cells from *n* = 4 mice). Very few (0.2 ± 0.2%) NeuN immunoreactive cells had mCherry immunoreactivity (1 of 509 cells from *n* = 4 mice). AP, area postrema; HGN, hypoglossal nucleus; NTS, nucleus of the solitary tract; X, dorsal motor nucleus of the vagus. Data are expressed as mean ± *SE* of the mean [Color figure can be viewed at http://wileyonlinelibrary.com]

It is established that Gq‐coupled DREADDs can be used to selectively activate astrocytes by driving increases in intracellular Ca^2+^ (Adamsky et al., 2018; Bonder & McCarthy, 2014; Chen et al., 2016; Durkee et al., 2019; Martin‐Fernandez et al., 2017). We bilaterally injected AAV vectors containing hM3Dq_mCherry (DVC::GFAP^hM3Dq^ mice) or mCherry (DVC::GFAP^mCherry^ mice) under the control of the GFAP promoter (GFAP) into the dorsal portion of the caudal brainstem with the goal of limiting expression to DVC astrocytes (Figure 2a,b). Examination of mCherry‐immunoreactivity indicated strong vector‐mediated transduction of the DVC (Figure 2c, Supplementary Figure S2a–c). Using double‐fluorescence immunohistochemistry, the proportion of GFAP‐immunoreactive astrocytes in which mCherry immunoreactivity was detected was quantified. Within the DVC, 91.49 ± 1.6% of GFAP expressing cells were mCherry positive (Figure 2d; 242 of 265 cells from *n* = 4 mice). In contrast, 0.2 ± 0.2% of DVC NeuN expressing cells were mCherry positive (Figure 2e; 1 of 509 cells from *n* = 4 mice). This indicates that within the DVC appropriate specificity of vector transduction of glia was achieved.

After CNO injection (0.3 mg/kg i.p.), GFAP‐expressing astrocytes in the postremal NTS of DVC::GFAP^hM3Dq^ mice had a greater morphological complexity and number of processes than those of DVC::GFAP^mCherry^ mice (Supplementary Figure S3). This indicates that in DVC::GFAP^hM3Dq^ mice CNO treatment was sufficient to induce changes in the structure of the DVC GFAP‐immunoreactive astrocytes, indicative of activation.

### 3.3 Chemogenetic activation of DVC astrocytes reduced food intake

**Figure 3:**
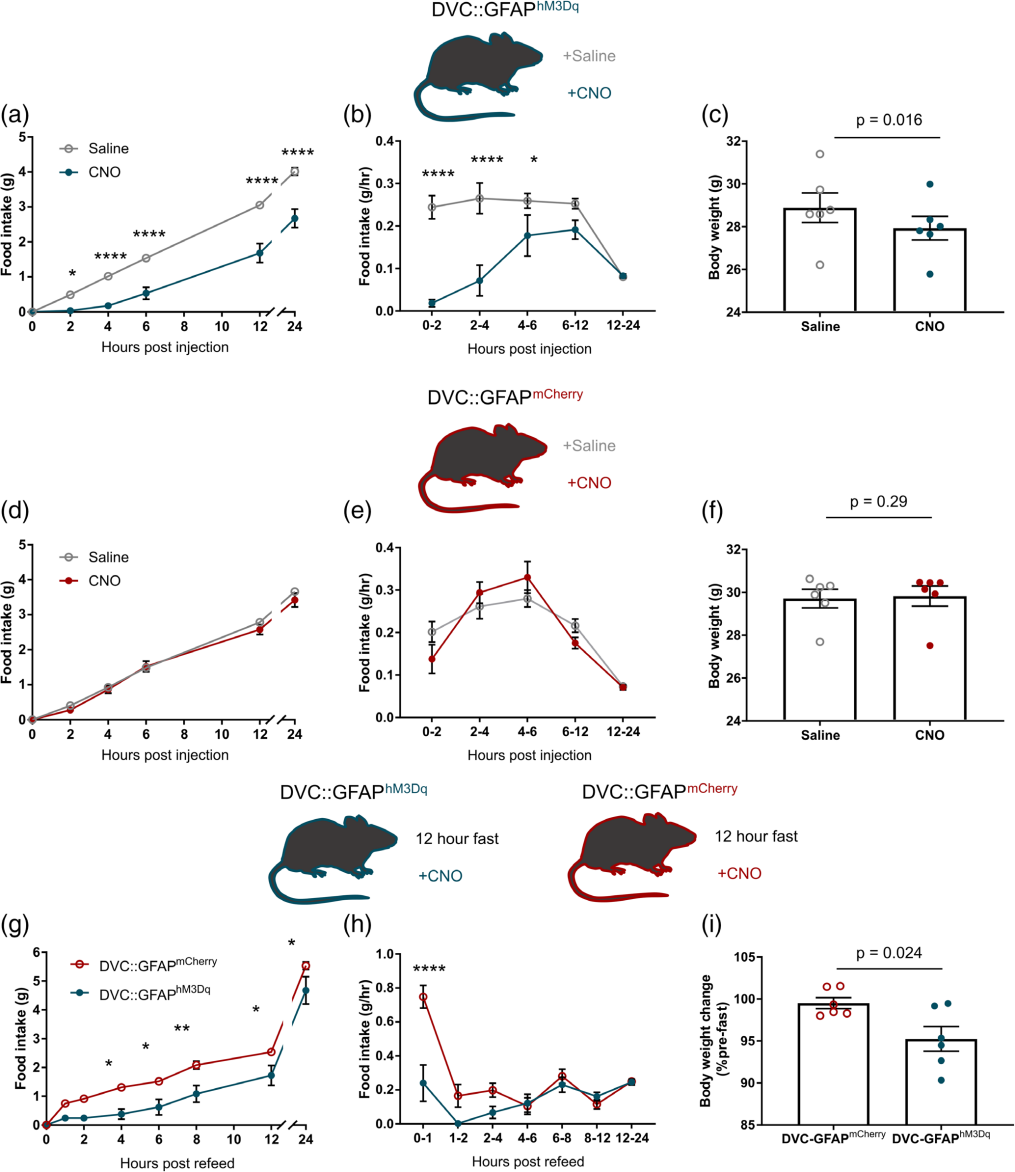
Chemogenetic activation dorsal vagal complex (DVC) astrocytes reduced food intake. (a–c) DVC::GFAP^hM3Dq^ mice (*n* = 6) were injected with saline or clozapine‐*N*‐oxide (CNO) 15–30 min prior to the beginning of the dark phase. (a) Cumulative food intake (two‐way repeated measure [RM] analysis of variance [ANOVA], CNO, *p* = .0007, *F*_(1,5)_ = 55.7; time, *p* < .0001, *F*_(5,25)_ = 331.3; interaction, *p* < .0001, *F*_(5,25)_ = 15.64, Sidak's post hoc test). (b) Rate of food intake (two‐way RM ANOVA, CNO, *p* = .0009, *F*_(1,5)_ = 49.15; time, *p* = .0001, *F*_(4,20)_ = 9.94; interaction, *p* < .0001, *F*_(4,20)_ = 11.07, Sidak's post hoc test). (c) Body weight 8 hr after lights‐off (28.89 ± 0.69 vs. 27.93 ± 0.55 g, *p* = .016, paired *t* test). (d–f) DVC::GFAP^mCherry^ mice (*n* = 6) were injected with saline or CNO 30 min prior to the beginning of the dark phase. (d) Cumulative food intake (two‐way RM ANOVA, CNO, *p* = .33, *F*_(1,5)_ = 1.15; time, *p* < .0001, *F*_(5,25)_ = 506.7; interaction, *p* = .18, *F*_(5,25)_ = 1.65, Sidak's post hoc test). (e) Rate of food intake (two‐way RM ANOVA, CNO, *p* = .76, *F*_(1,5)_ = .10; time, *p* < .0001, *F*_(4,20)_ = 29.57; interaction, *p* = .07, *F*_(4,20)_ = 2.56, Sidak's post hoc test). (f) Body weight 8 hr after lights‐off (29.59 ± 0.43 vs. 29.83 ± 0.47 g, *p* = .29, paired *t* test). (g–i) DVC::GFAP^mCherry^ and DVC::GFAP^hM3Dq^ mice (*n* = 6/group) were fasted for 12 hr during the dark phase then injected with CNO 30 min prior to reintroduction of food at the onset of the light phase. (g) Cumulative food intake (two‐way ANOVA, DREADD, *p* = .01, *F*_(1,10)_ = 10.03; time, *p* < .0001, *F*_(7,70)_ = 310.5; interaction, *p* = .0053, *F*_(7,70)_ = 3.20, Sidak's post hoc test). (h) Rate of food intake (two‐way ANOVA, DREADD, *p* = .007, *F*_(1,10)_ = 11.4; time, *p* < .0001, *F*_(6,60)_ = 18.55; interaction, *p* < .0001, *F*_(6,60)_ = 8.35, Sidak's post hoc test). (i) Body weight (% of prefasting weight) 8 hr after refeeding (99.51 ± 0.65 vs. 95.24 ± 1.47%, *p* = .024 unpaired *t* test). *p < .05, **p < .01, ****p < .0001. Data are expressed as mean ± *SE* of the mean [Color figure can be viewed at http://wileyonlinelibrary.com]

Mice typically eat most of their food during the dark‐phase. Chemogenetic activation of the transduced astrocytes in DVC::GFAP^hM3Dq^ mice (CNO; 0.3.mg/kg, i.p. 15–30 min prior to onset of the dark‐phase) produced an 84% reduction in food intake (4 hr after injection) compared to food consumption by the same mice following saline control treatment (Figure 3a). This anorectic effect lasted for approximately 6 hr after which the rate of food intake returned to that seen following saline treatment (Figure 3b). Body weight, measured 8 hr after lights‐off, was reduced by 4.4% on the day of CNO injection compared with the day of saline injection (Figure 3c). In DVC::GFAP^mCherry^ mice, the same CNO injection protocol had no effect on dark‐phase food intake, feeding rate, or body weight (Figure 3d–f). Following injection of CNO (0.3 mg/kg i.p.), both food intake and food seeking behavior were lower in DVC::GFAP^hM3Dq^ mice compared with DVC::GFAP^mCherry^ mice (Supplementary Figure S4a–c). This suggests that the reduced food intake is a result of suppressed drive to feed rather than reflecting a motor impairment of feeding, such as could be anticipated as a result of activation of astrocytes in the hypoglossal nucleus (HGN) disrupting tongue movement.

To evaluate whether chemogenetic activation of DVC astrocytes was sufficient to reduce feeding when there was an increased drive to eat, we utilized a fast‐induced refeeding paradigm. CNO injection (0.3 mg/kg i.p.) 15–30 min prior to reintroduction of food after a 12 hr fast lowered cumulative food intake during the refeeding phase in DVC::GFAP^hM3Dq^ mice compared with DVC::GFAP^mCherry^ controls (Figure 3g). During the first hour of refeeding, the rate of food intake was 63% lower in DVC::GFAP^hM3Dq^ mice, compared with DVC::GFAP^mCherry^ controls, indicating that chemogenetic activation of DVC astrocytes was sufficient to acutely suppress the drive to eat induced by fasting (Figure 3h). No compensatory/rebound hyperphagia was observed in DVC::GFAP^hM3Dq^ mice in the 24 hr following CNO injection (Figure 3g,h). In line with the differences in food intake, within 8 hr of food being reintroduced DVC::GFAP^mCherry^ mice recovered more of their body weight lost as a result of the fast than DVC::GFAP^hM3Dq^ mice (Figure 3i).

Following viral injection, although some glia were also transduced in surrounding nuclei (Supplementary Figure S2a–c), linear regression analysis revealed no relationship between the size of the transduced area and the effect of CNO injection on food intake in the DVC::GFAP^hM3Dq^ mice (Supplementary Figure S2d). Since the DVC (Bregma −7.48 mm) was transduced in all these mice this suggests that the transduction of neighboring nuclei is unlikely to account for the effect on food intake. Furthermore, the suppressive effect of CNO on both dark‐phase and fast‐induced refeeding in DVC::GFAP^hM3Dq^ mice (compared DVC::GFAP^mCherry^ mice) was replicated in a second cohort of mice who had a more discrete vector injection: bilateral injections at only one site in the DVC (Supplementary Figure S5). These findings indicate that it is the glia within the DVC that mediate the suppression of feeding.

### 3.4 Chemogenetic activation of DVC astrocytes did not alter locomotion

**Figure 4:**
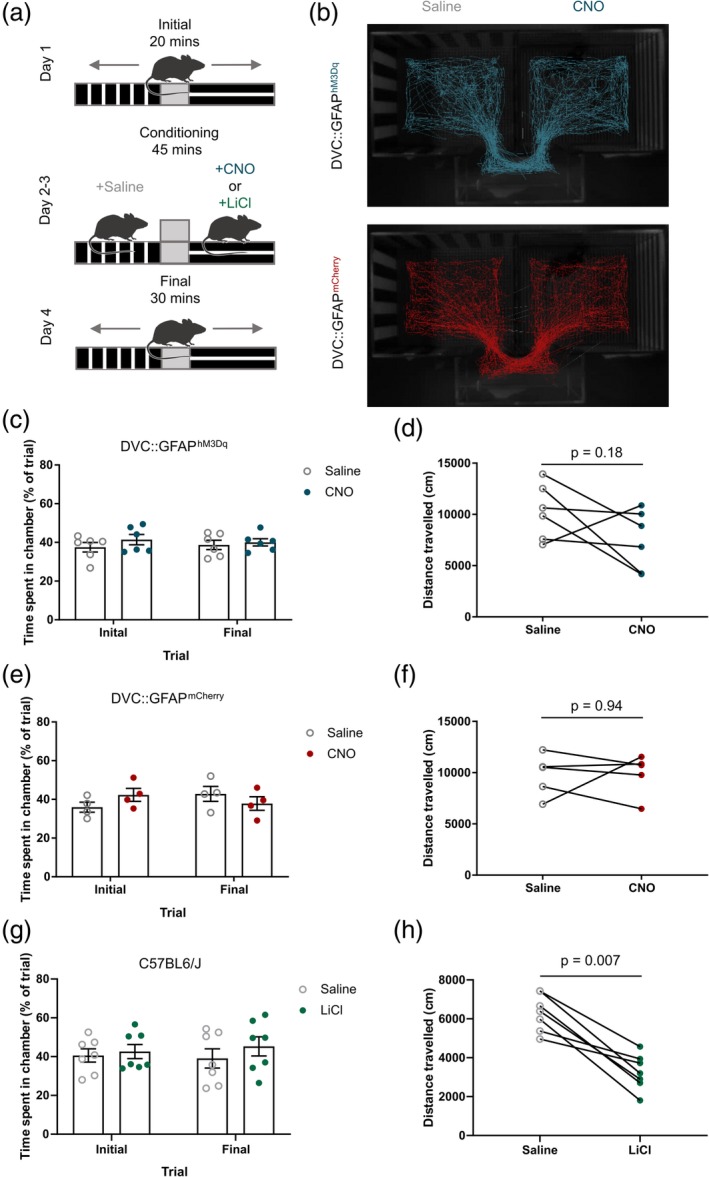
Injection with clozapine‐*N*‐oxide (CNO) did not affect locomotion in DVC::GFAP^hM3Dq^ or DVC::GFAP^mCherry^ mice. (a) Schematic of the conditioning protocol. (b) Representative tracks of a DVC::GFAP^hM3Dq^ (top, blue) and a DVC::GFAP^mCherry^ (bottom, red) mouse during the final trial. (c) Percentage of the trial spent in the saline‐ and CNO‐paired chamber before (initial) and after (final) conditioning sessions in DVC::GFAP^hM3Dq^ mice (*n* = 6 mice, two‐way repeated measure [RM] analysis of variance [ANOVA], CNO, *p* = .32, *F*_(1,10)_ = 1.09; conditioning, *p* = .96, *F*_(1,10)_ = 0.002; interaction, *p* = .57, *F*_(1,10)_ = 0.35). (d) Distance travelled during conditioning sessions for DVC::GFAP^hM3Dq^ mice (*n* = 6 mice; 10,260 ± 1,101 vs. 7,501 ± 1,183 cm; *n* = 6 mice; *p* = .18, paired *t* test). (e) Percentage of the trial spent in the saline‐ and CNO‐paired chamber before and after conditioning sessions in DVC::GFAP^mCherry^ mice (*n* = 4 mice, two‐way RM ANOVA, CNO, *p* = .76, *F*_(1,6)_ = 0.10; conditioning, *p* = .78, *F*_(1,6)_ = 0.08; interaction, *p* = .23, *F*_(1,6)_ = 1.81). (f) Distance travelled during conditioning sessions for DVC::GFAP^mCherry^ mice (*n* = 5 mice; 9,773 ± 991.1 vs. 9,870 ± 896.3 cm; *n* = 5 mice; *p* = .94; paired *t* test). (g) Percentage of the trial spent in the saline‐ and LiCl‐paired chamber before and after conditioning sessions (*n* = 7 mice, two‐way RM ANOVA, LiCl, *p* = .48, *F*_(1,12)_ = 0.54; Conditioning, *p* = .8, *F*_(1,12)_ = 0.06; interaction, *p* = .38, *F*_(1,12)_ = 0.82). (h) Distance travelled during conditioning sessions (6,316 ± 360.9 vs. 3,261 ± 343.4 cm; *n* = 7 mice; *p* = .007; paired *t* test). Data are expressed as mean ± *SE* of the mean [Color figure can be viewed at http://wileyonlinelibrary.com]

Reductions in food intake in mice can be indicative of malaise and/or aversion (Maniscalco & Rinaman, 2018). To test whether DVC astrocyte activation was aversive, we used a CPA assay (Figure 4a). During initial assessment, prior to conditioning, neither DVC::GFAP^hM3Dq^ nor DVC::GFAP^mCherry^ mice showed a preference for either chamber (Figure 4c,e). After conditioning (pairing each side of the apparatus with either saline or CNO injection [0.3 mg/kg i.p.]), mice again showed no preference or aversion (Figure 4b,c,e). However, following the same conditioning protocol using LiCl (150 mg/kg i.p.), a known aversive stimulus, an independent cohort of mice showed no avoidance of the LiCl chamber (Figure 4g); as such, from our experiment, the effect of DVC astrocyte activation on conditioned aversion is unclear.

The conditioning protocol did however allow measurement of locomotion following chemogenetic DVC astrocyte activation. There was no statistically significant difference in the total distance travelled during the 45 min conditioning trial following saline or CNO treatment the DVC::GFAP^hM3Dq^ or DVC::GFAP^mCherry^ groups (Figure 4d,f). This suggests that CNO‐mediated DREADD activation did not impact locomotion. This is in contrast to mice treated with LiCl which displayed a statistically significant reduction in total distance travelled, when compared with the saline conditioning trial (Figure 4h). This indicates that at the dose used LiCl is sufficient to acutely attenuate locomotion in mice, potentially indicative of malaise, while either chemogenetic activation of DVC astrocytes or CNO treatment in DVC::GFAP^mCherry^ mice is not.

### 3.5 Chemogenetic activation of DVC astrocytes induced c‐FOS expression locally and in the lPBN

Immunoreactivity for the immediate early gene product c‐FOS, as a marker of cellular activation, was assessed to quantitate the extent of DVC activation and to identify possible downstream neuronal circuit engagement. DVC::GFAP^hM3Dq^ and DVC::GFAP^mCherry^ mice were injected with CNO 2–3 hr prior to perfusion fixation followed by immunohistochemistry for c‐FOS.

**Figure 5:**
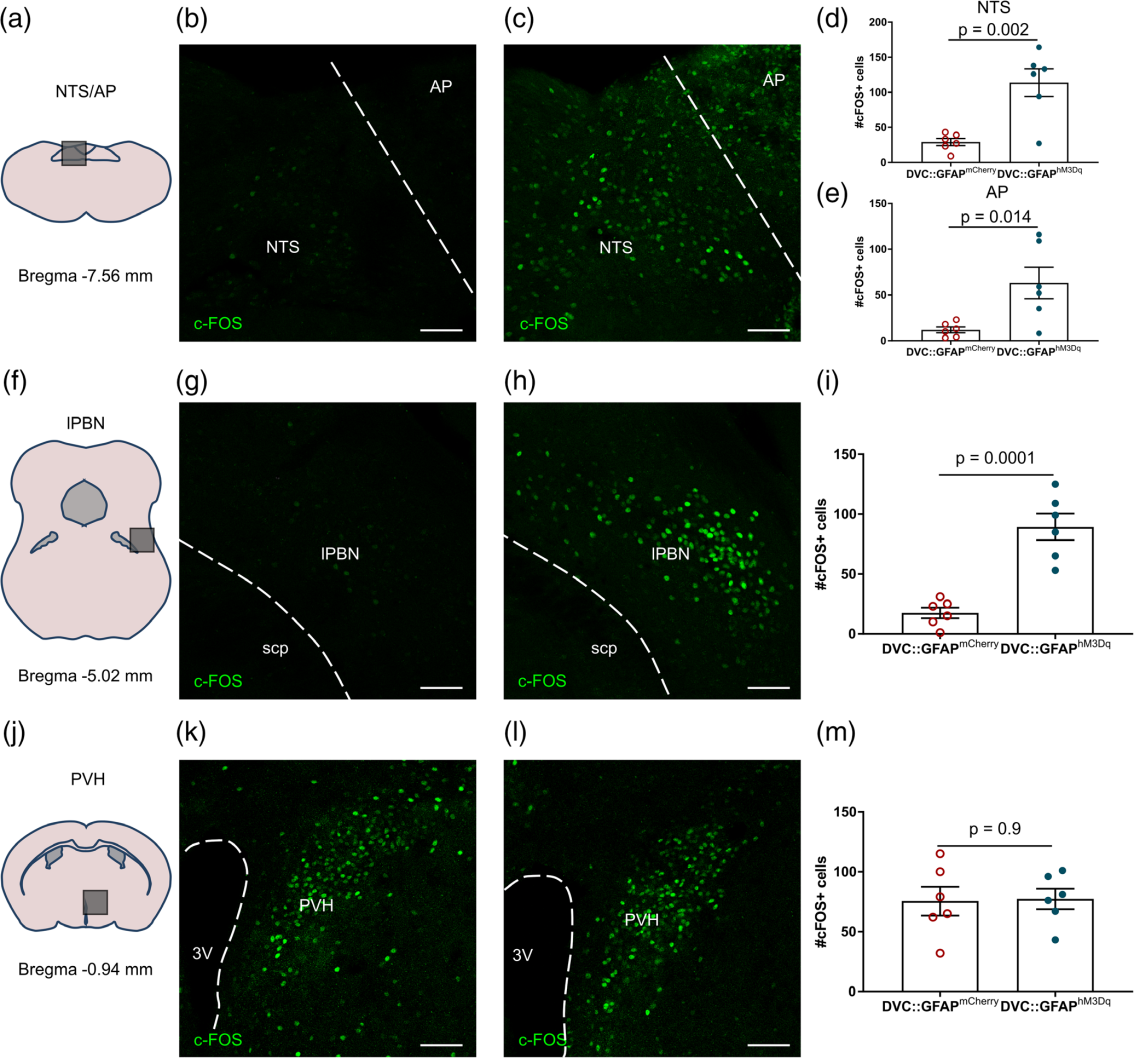
Chemogenetic astrocyte activation induced c‐FOS‐immunoreactivity in brainstem neural circuits in DVC::GFAP^hM3Dq^ mice. DVC::GFAP^mCherry^ and DVC::GFAP^hM3Dq^ mice (*n* = 6 mice/group) were injected with CNO 2–3 hr prior to perfusion‐fixation. (a–c) Representative images of c‐FOS immunostaining from the dorsal vagal complex (DVC) of a DVC::GFAP^mCherry^ mouse (b) and a DVC::GFAP^hM3Dq^ mouse (c). (d,e) Quantification of c‐FOS immunoreactive cells in the nucleus of the solitary tract (NTS) (d) (29 ± 5.01 vs. 113.7 ± 19.62 cells, *p* = .002, unpaired *t* test) and AP (e) (11.83 ± 3.2 vs. 63.17 ± 17.2 cells, *p* = .014, unpaired *t* test). (f–h) Representative images of c‐FOS immunostaining from the lateral parabrachial nucleus (lPBN) of a DVC::GFAP^mCherry^ mouse (g) and a DVC::GFAP^hM3Dq^ mouse (h). (i) Quantification of c‐FOS immunoreactive cells in lPBN (17.5 ± 4.49 vs. 89.33 ± 11.08 cells, *p* = .002, unpaired *t* test). (j–l) Representative images of c‐FOS immunostaining from the lPBN of a DVC::GFAP^mCherry^ mouse (k) and a DVC::GFAP^hM3Dq^ mouse (l). (m) Quantification of c‐FOS immunoreactive cells in paraventricular nucleus of the hypothalamus (PVH; 75.5 ± 12.05 vs. 77.33 ± 8.58 cells, *p* = .90, unpaired *t* test). For all images, scale bar = 50 μm. AP, area postrema; lPBN, lateral parabrachial nucleus; NTS, nucleus of the solitary tract; PVH, paraventricular nucleus of the hypothalamus; scp, superior cerebellar peduncle. Data are expressed as mean ± *SE* of the mean [Color figure can be viewed at http://wileyonlinelibrary.com]

Chemogenetic activation (CNO 0.3 mg/kg i.p.) of DVC astrocytes increased c‐FOS expression in the NTS and AP (Figure 5a–e), and the lPBN, a downstream target of NTS neurons (Figure 5f–i) in DVC::GFAP^hM3Dq^ mice compared with DVC::GFAP^mCherry^ control mice. However, in the PVH, another projection target of satiety signaling NTS neurons (D'Agostino et al., 2016; Roman, Sloat, & Palmiter, 2017), there was no difference in the number of c‐FOS expressing cells between groups (Figure 5j–m). This is consistent with DVC astrocyte activation signaling to neuronal circuitry implicated in regulation of energy homeostasis (Atasoy, Betley, Su, & Sternson, 2012; Carter, Soden, Zweifel, & Palmiter, 2013).

## 4 DISCUSSION

In this study, we have shown for the first time that astrocytes in the NTS react dynamically to excess intake of an energy dense food by upregulating GFAP expression and showing morphological plasticity. Furthermore, DREADD‐mediated activation of astrocytes in the DVC caused a potent but reversible decrease in food intake, associated changes in NTS astrocyte morphology, and activation of local and distal neuronal circuits. This evidence supports the hypothesis that DVC astrocytes are involved in a homeostatic or allostatic response to nutritional excess, and their activation may serve to drive a counter‐acting decrease in food intake to restore energy balance.

Astrocytes of the ARC have previously been implicated in the regulation of food intake (Buckman et al., 2015). In the hypothalamus, these glia are responsive to short‐term energy imbalance, both fasting and acute high‐fat feeding (Buckman et al., 2015; Fuente‐Martín et al., 2012), and direct manipulation of their activity using DREADDs alters food intake (Chen et al., 2016; Yang et al., 2015). Both studies using activating DREADDs in ARC astrocytes show a modest increase in food intake during the light phase, when mice are not typically eating (Chen et al., 2016; Yang et al., 2015). In contrast, it appears from our observations that chemogenetic DVC astrocyte activation has more pronounced effects on feeding with respect to the magnitude, direction, and onset/duration of the response. The less pronounced impact of chemogenetic modulation of ARC astrocytes on feeding behavior in mice may be due, in part, to the fact that the ARC contains at least two neurochemically unique neuronal populations: AgRP and proopiomelanocortin (POMC) neurons; the activation of which drives and inhibits feeding behavior, respectively (Aponte, Atasoy, & Sternson, 2011; Krashes et al., 2011). As the two neuronal populations are anatomically interspersed yet functionally opposite, chemogenetic activation of ARC astrocytes may result in modulation of the activity of both neuronal populations making interpretation of the physiological impact on feeding complex. With respect to feeding, to date, neurons of the NTS have principally been implicated in satiety rather than hunger (Grill & Hayes, 2012), which may account for the more pronounced physiological effect consequent on local astrocytic activation.

Our study builds upon evidence showing that astrocytes of the NTS sense hormonal satiety signals (Reiner et al., 2016) and are involved in integration of vagal neurotransmission (McDougal et al., 2011), but importantly extends these studies by using direct, specific, and inducible activation of these cells to study behavior in freely moving animals. What remains unclear are the mechanisms by which astrocytes are activated in the state of caloric excess. Likely candidates include vagal‐derived glutamate (McDougal et al., 2011), GLP‐1 mediated signaling (Reiner et al., 2016), and/or local inflammation caused by circulating saturated fatty acids or neurogenic neuroinflammation (Xanthos & Sandkühler, 2014). These factors may act in combination to promote changes in NTS astrocyte function in the face of nutritional perturbation.

In this study, we used morphological complexity as a marker of altered astrocyte signaling to demonstrate that NTS astrocytes were responsive to both acute high‐fat feeding and chemogenetic stimulation. A higher baseline of GFAP expression was observed in animals that had undergone AAV injection surgery. The morphological analysis of NTS astrocytes presented in this study encompassed the proximal processes of GFAP‐expressing astrocytes. While this may represent only a small percentage (~10%) of the total astrocyte surface area (Shigetomi et al., 2013), there was a significant difference in the Sholl profile and number of processes between groups supporting a reactive change in astrocyte structure under these experimental conditions. Other strategies which label a greater proportion of the astrocyte cell volume (e.g., Golgi staining or membrane targeted green fluorescent protein [lck‐GFP]) could be used for future studies but each has their own limitations: Golgi staining does not positively differentiate between astrocytes and neurons and sparsely labels cells, while lck‐GFP (which labels the entire astrocyte surface area) yields a “bushy” signal (Shigetomi et al., 2013), that is not resolvable by standard confocal microscopy.

We found that CNO treatment in DVC::GFAP^hM3Dq^ mice decreased food intake and induced c‐FOS expression in neighboring neurons; however, the underlying molecular mechanisms by which this occurs remain to be resolved. Astrocytes can modulate the activity of neurons by mechanisms including altered glutamate transport and also via release of neuroactive molecules (e.g., glutamate, ATP, d‐serine) (Araque et al., 2014; Gourine et al., 2010; Matott, Kline, & Hasser, 2017; Panatier et al., 2006; Papouin, Dunphy, Tolman, Dineley, & Haydon, 2017; Schwarz, Zhao, Kirchhoff, & Bruns, 2017). Critically, glutamatergic signaling is the principal mechanism for communication between the vagus and second‐order NTS neurons (Doyle & Andresen, 2001) and NTS astrocytes directly sense vagal glutamate release via Ca^2+^‐permeable AMPA receptors expressed on the cell membrane (McDougal et al., 2011). In the NTS, synaptic clearing of glutamate by astrocytes has been shown to restrain NTS neuronal firing and vagal outflow to cardiorespiratory organs (Matott et al., 2017). As such, astrocyte glutamate transport can manipulate the firing rates of NTS neurons and alter output from the NTS. In our study, it is unclear how (and indeed if) astrocyte Gq‐GPCR activation may be linked to altered glutamate uptake; however, previous reports (Oliet, Piet, & Poulain, 2001) have shown that altered morphology of astrocytes results in corresponding changes in glutamate uptake and neuronal excitability. An additional potential mechanism of communication that could be important in mediating the effects seen is active gliotransmission (Araque et al., 2014). Activation of a GPCR expressed on NTS astrocytes (protease‐activated receptors) leads to increased activation of neurons by glutamate, possibly exocytosed by astrocytes (Vance, Rogers, & Hermann, 2015). Given that antagonism of NMDA receptors in the NTS increases meal size (Treece, Covasa, Ritter, & Burns, 1998), it is possible that activation of these receptors by astrocyte‐derived glutamate or d‐serine would reduce food intake, but further study would be required to specifically test this.

Our data support the conclusions of an earlier study which showed that DVC astrocytes are critical for the appetite suppressive effect of GLP‐1 receptor (GLP‐1R) activation. In rats, NTS astrocytes take up exendin‐4 (an agonist of the GLP‐1R) and metabolic inhibition of astrocytes in the DVC using fluorocitrate abolishes the anorexigenic actions of this compound. Together, this suggests that astrocytes contribute to the suppressive effect of activation of GLP1‐R signaling on feeding and supports their role in DVC‐mediated satiety signaling (Reiner et al., 2016). What remains unclear is whether this is due to removal of GLP‐1R‐mediated astrocyte activation or alternatively astrocyte inhibition causing disruption to glutamatergic signaling stimulated by neuronal GLP‐1R activation. While fluorocitrate has historically been widely used to inhibit glial cell activity, it would be beneficial to repeat these studies using genetically targeted approaches to modulate astrocyte activity. Whether GLP‐1R is expressed in astrocytes is also not clear as GFP expression was not apparent in DVC astrocytes of GLP‐1R‐GFP mice (Cork et al., 2015). However, in vitro and in ex vivo rat brainstem slices astrocytes increase intracellular Ca^2+^ in response to exendin‐4 providing evidence that these cells are directly sensitive to GLP‐1R ligands (Marina et al., 2017; Reiner et al., 2016).

It is likely that DVC astrocytes are important for integration of hormonal and nutritional cues. Hypothalamic astrocytes express receptors for ghrelin, insulin, and leptin (Fuente‐Martín et al., 2016; García‐Cáceres et al., 2016; Kim et al., 2014). In common with the ARC, the DVC contains a circumventricular site, the AP, making it likely that DVC astrocytes also share the same hormone sensing capabilities, although this remains to be conclusively demonstrated. Reexpression of the glucose transporter GLUT2 in astrocytes is sufficient to restore brainstem hypoglycemia detection in GLUT2‐knockout mice providing further evidence for a critical role of these cells in energy homeostasis (Marty et al., 2005).

Of note, three studies that inhibit inflammatory nuclear factor kappa b (NF‐κB) signaling in all GFAP‐expressing astrocytes (including work from our group) report feeding phenotypes, namely, increased initial intake of a high‐fat diet following initial exposure, resistance to the obesity phenotype when already on a high‐fat diet and protection from metabolic dysfunction and weight gain on a high fat diet (Buckman et al., 2015; Douglass, Dorfman, Fasnacht, Shaffer, & Thaler, 2017; Zhang et al., 2017). Although these studies attribute the observed effects to astrocytes of the hypothalamus the contribution of NTS astrocytes, identified here as responsive to high‐fat diet intake, cannot be ruled out.

The induction of c‐FOS‐immunoreactivity in a distal target, namely, the lPBN, following CNO injection in DVC::GFAP^hM3Dq^ mice strongly suggests the recruitment of long‐range neuronal circuitry following DVC astrocyte activation. Since there are a number of distinct neuronal populations in the NTS whose activation induces a similar reduction in feeding, namely, POMC (Cerritelli et al., 2016; Zhan et al., 2013), cholecystokinin (CCK) (D'Agostino et al., 2016; Roman et al., 2016), tyrosine hydroxylase (Roman et al., 2016), and preproglucagon (Gaykema et al., 2017), it is likely that these are among the neurons recruited by chemogenetic DVC astrocyte activation.

Given the reduction in food intake we observed, it was important to evaluate whether induction of malaise and/or aversion could be contributing to the effect seen, particularly as the DVC is implicated in mediating these responses in addition to satiety (Maniscalco & Rinaman, 2018). This particularly important in light of the increased in c‐FOS immunoreactivity observed in the lPBN of DVC::GFAP^hM3Dq^ mice after CNO treatment, which may indicate the activation of a NTS‐lPBN pathway previously reported to reduce food intake through aversion/negative salience (Roman et al., 2017). To do this, we used a CPA assay utilizing a protocol previously used by our group to indicate aversive responses/negative salience in response to chemogenetic activation of prefrontal cortex‐projecting locus coeruleus neurons (LC^PFC^) in rats (Hirschberg, Li, Randall, Kremer, & Pickering, 2017). We found that after one conditioning session per agent CNO treatment in DVC::GFAP^hM3Dq^ or DVC::GFAP^mCherry^ mice did not induce CPA. However, since the known aversive LiCl agent was unable to induce CPA in this paradigm it suggests our assay was likely not sufficiently sensitive to detect/induce CPA in this context. As such, the affective properties of DREADD‐mediated DVC astrocyte activation remain unclear. A longer protocol may have revealed an effect since three or four conditioning sessions with LiCl does induce CPA in mice (Le Merrer et al., 2011; Longoni, Spina, Vinci, & Acquas, 2011; Sanjakdar et al., 2015; Zhang et al., 2019). We did, however, find that LiCl injection reduced locomotion during the conditioning trial while CNO treatment did not. This suggests LiCl acutely induces malaise; an effect that was not seen in the CNO‐treated mice. Evidence suggests that satiety and aversion exist on a continuum, where in some cases aversion may be a manifestation of extreme satiety, for example, associated with excessive gastrointestinal distention (Maniscalco & Rinaman, 2018). As such, ability of an agent to induce CPA or conditioned taste aversion does not necessarily indicate that an effect is non‐physiological. For example, exogenous CCK can induce satiety or aversion dependent on dose (Moran, 2000; Swerdlow, van der Kooy, Koob, & Wenger, 1983; West, Greenwood, Marshall, & Woods, 1987). Our results suggest that that DREADD‐mediated DVC astrocyte activation likely does not produce acute malaise, but its emotional salience remains unclear and further studies would be required to explore this.

In conclusion, our experiments show that astrocytes may be a previously overlooked element of DVC‐mediated satiety and provide the first causal link between their activity and the regulation of feeding. We propose that NTS/DVC astrocytes are a key component in satiety signaling that act to modulate the integration of vagal and hormonal inputs to the brainstem and as such could represent a potential target for intervention in obesity.

## CONFLICT OF INTEREST

The authors declare no potential conflict of interest.

## Supporting information


**Supplementary Figure S1 | Demonstration of specificity of antibody binding.** Representative images of sections stained in the absence of the primary antibody. **a,** donkey anti‐mouse Alexa Fluor 568 **b,** donkey anti‐mouse Alexa Fluor 488 **c**, donkey anti‐rabbit Alexa Fluor 488. Same section showing DAPI staining to right. Scale bar = 50 μm. AP = area postrema, NTS = nucleus of the solitary tract.
**Supplementary Figure S2 | AAV vector spread in individual DVC::GFAP** ^**hM3Dq**^ **mice.** Visualization of mCherry reporter protein allowed mapping of the extent of transduction resulting from injection. **a‐c,** Shown are the schematic diagrams of the patterns of mCherry expression and representative images from DVC:GFAP^hM3Dq^ mice (n = 6) at Bregma −7.92 mm **(a)**, Bregma −7.48 mm **(b)** and Bregma −7.08 mm **(c)**. Transduced regions are shown overlaid (darkest where all 6 mice overlap). **d,** Relationship between transduced area of postremal section (Bregma −7.48 mm) and the CNO‐induced reduction of food intake (calculated as control food intake [saline] minus CNO food intake 4 hr post injection) (Linear regression slope = −0.04, not significantly different from zero *p* = 0.93). Scale bar = 500 μm. 4 V = fourth ventricle, AP = area postrema, NTS = nucleus of the solitary tract, X = dorsal motor nucleus of the vagus.
**Supplementary Figure S3 | Chemogenetic activation of DVC astrocytes increased morphological complexity.** DVC::GFAP^mCherry^ and DVC::GFAP^hM3Dq^ mice were injected with CNO (0.3 mg/kg) and perfused 2–3 hr later. **a,b,** Representative confocal image of GFAP immunostaining from a DVC::GFAP^mCherry^ **(a)** and a DVC::GFAP^hM3Dq^ **(b)** mouse, scale bar = 50 μm. **c,** Mean Sholl profile of postremal NTS astrocytes of DVC::GFAP^mCherry^ and DVC::GFAP^hM3Dq^ mice (n = 35–40 cells from 4 mice/group, Two‐way ANOVA, DREADD, *p* < 0.0001, F_(1,1,440)_ = 63.05; Distance from soma, p < 0.0001, F_(19,1,440)_ = 167.0; interaction, p = <0.0001, F_(19,1,440)_ = 3.49; Sidak's post hoc test). **d,** Number of processes of individual postremal NTS astrocytes of DVC::GFAP^mCherry^ and DVC::GFAP^hM3Dq^ mice (6.14 ± 029 vs 7.88 ± 0.43, n = 35–40 cells from 4 mice/group, *p* = 0.0015, unpaired *t test*). **e,f** Representative image and trace of a GFAP+ cell from a DVC::GFAP^mCherry^ **(e)** and a DVC::GFAP^hM3Dq^ **(f)** mouse, scale bar = 25 μm. * = *p* < 0.05, ** = *p* < 0.01, **** = *p* < 0.0001. Data are expressed as mean ± standard error of the mean.
**Supplementary Figure S4 | Food seeking was lower in DVC::GFAP** ^**hM3Dq**^ **than DVC::GFAP** ^**mCherry**^ **mice following CNO injection.** DVC::GFAP^mCherry^ and DVC::GFAP^hM3Dq^ mice (n = 3 mice/group) were injected with CNO at the beginning of the dark‐phase and video monitored for 3 hr in their home cage with food pellets in the far corner from their nest. **a,** Food intake during the 3 hr monitoring period (0.79 ± 0.10 vs 0.21 ± 0.14 g, *p* = 0.028, unpaired *t test*). **b,** Total number of entries to the food containing zone of the cage made during the 3 hr monitoring period (118.3 ± 12 vs 30.67 ± 15.38 entries, *p* = 0.011, unpaired t test). **c,** Number of entries to the food containing zone of the cage in ten minute bins made by DVC::GFAP^mCherry^ (red) and DVC::GFAP^hM3Dq^ (blue) mice in the first hr of the monitoring period (Two‐way ANOVA, DREADD, *p* = 0.0052, F_(1,4)_ = 30.81; Time, *p* < 0.0019, F_(5,20)_ = 5.74; interaction, *p* < 0.013, F_(5,20)_ = 3.85, Sidak's post hoc test). **d,** relationship between total food intake and total entries to the food containing zone (linear regression slope = 141.8, significantly non‐zero *p* = 0.0002). **** = *p* < 0.0001. Data are expressed as mean ± standard error of the mean.
**Supplementary Figure S5 | Single AAV injection to DVC gave the same feeding suppressive effect. a,** AAV vectors containing either hM3Dq_mCherry or mCherry under the hGFAP promoter. **b,** Schematic of bilateral injection of the vector into the DVC. **c,** Representative image showing mCherry immunofluorescence in a DVC::GFAP^hM3Dq^ mouse, scale bar = 500 μm. **d, e,** DVC::GFAP^hM3Dq^ mice (previously transduced with a 180 nL single vector injection bilaterally, n = 4) were injected i.p. with saline or CNO (0.3 mg/kg) 30 minutes prior to the beginning of the dark‐phase. **d,** Cumulative food intake (Two‐way RM ANOVA, CNO, *p* < 0.0001, F_(1,18)_ = 335.1; Time, *p* < 0.0001, F_(5,18)_ = 112.0; interaction, p < 0.0001, F_(5,18)_ = 18.64, Sidak's post hoc test). **e,** Rate of food intake (n = 4 mice, Two‐way RM ANOVA, CNO, *p* = 0.0036, F_(1,3)_ = 69.45; Time, *p* = 0.0019, F_(4,12)_ = 8.25; interaction, p < 0.0001, F_(4,12)_ = 16.03, Sidak's post hoc test). **f, g,** DVC::GFAP^mCherry^ and DVC::GFAP^hM3Dq^ mice (n = 3‐4/group) were fasted for 12 hr during the dark phase then injected i.p. with CNO (0.3 mg/kg) 30 minutes prior to reintroduction of food at the onset of the light phase. **f,** Cumulative food intake (Two‐way ANOVA, DREADD, *p* = 0.038, F_(1,5)_ = 7.818; Time, *p* < 0.0001, F_(7,35)_ = 168.0; interaction, *p* = 0.026, F_(7,35)_ = 2.67, Sidak's post hoc test). **g,** Rate of food intake (Two‐way ANOVA, DREADD, *p* = 0.029, F_(1,5)_ = 9.29; Time, *p* = 0.0001, F_(6,30)_ = 6.73; interaction, p < 0.0001, F_(6,30)_ = 11.21, Sidak's post hoc test). *p < 0.05, **p < 0.01, ****p < 0.0001. Data are expressed as mean ± standard error of the mean.

## Data Availability

The data that support the findings of this study are available from the corresponding author upon reasonable request.
