# Supplementary material for: Regulation of food intake by astrocytes in the brainstem dorsal vagal complex
Source: Glia. 2019 Dec 27;68(6):1241–54. doi: 10.1002/glia.23774 (PMC7187409; doi:10.1002/glia.23774)
Supplement: Supplementary file 1 — Supplementary Figure S1 | Demonstration of specificity of antibody binding. Representative images of sections stained in the absence of the primary antibody. a, donkey anti‐mouse Alexa Fluor 568 b, donkey anti‐mouse Alexa Fluor 488 c, donkey anti‐rabbit Alexa Fluor 488. Same section showing DAPI staining to right. Scale bar = 50 μm. AP = area postrema, NTS = nucleus of the solitary tract. Supplementary Figure S2 | AAV vector spread in individual DVC::GFAP hM3Dq mice. Visualization of mCherry reporter protein allowed mapping of the extent of transduction resulting from injection. a‐c, Shown are the schematic diagrams of the patterns of mCherry expression and representative images from DVC:GFAPhM3Dq mice (n = 6) at Bregma −7.92 mm (a), Bregma −7.48 mm (b) and Bregma −7.08 mm (c). Transduced regions are shown overlaid (darkest where all 6 mice overlap). d, Relationship between transduced area of postremal section (Bregma −7.48 mm) and the CNO‐induced reduction of food intake (calculated as control food intake [saline] minus CNO food intake 4 hr post injection) (Linear regression slope = −0.04, not significantly different from zero p = 0.93). Scale bar = 500 μm. 4 V = fourth ventricle, AP = area postrema, NTS = nucleus of the solitary tract, X = dorsal motor nucleus of the vagus. Supplementary Figure S3 | Chemogenetic activation of DVC astrocytes increased morphological complexity. DVC::GFAPmCherry and DVC::GFAPhM3Dq mice were injected with CNO (0.3 mg/kg) and perfused 2–3 hr later. a,b, Representative confocal image of GFAP immunostaining from a DVC::GFAPmCherry (a) and a DVC::GFAPhM3Dq (b) mouse, scale bar = 50 μm. c, Mean Sholl profile of postremal NTS astrocytes of DVC::GFAPmCherry and DVC::GFAPhM3Dq mice (n = 35–40 cells from 4 mice/group, Two‐way ANOVA, DREADD, p < 0.0001, F(1,1,440) = 63.05; Distance from soma, p < 0.0001, F(19,1,440) = 167.0; interaction, p = <0.0001, F(19,1,440) = 3.49; Sidak's post hoc test). d, Number of processes of individual postremal NTS [file GLIA-68-1241-s001.docx]

# Regulation of food intake by astrocytes in the brainstem dorsal vagal complex

**Running title:** Brainstem astrocytes regulate feeding

**Authors:** Alastair J. MacDonald^1,2^, Fiona E. Holmes^2^, Craig Beall^1^, Anthony E. Pickering^2,3^, Kate L.J. Ellacott^1,^*

**Institutional affiliations:**

1. Institute of Biomedical & Clinical Sciences, University of Exeter Medical School, RILD Building, Barrack Road, Exeter, EX2 5DW, United Kingdom
2. School of Physiology, Pharmacology and Neuroscience, University of Bristol, Biomedical Sciences Building, University Walk, Bristol, BS8 1TD, United Kingdom
3. Anaesthesia, Pain and Critical Care Sciences, Translational Health Sciences, Bristol Medical School, University of Bristol, Bristol, United Kingdom.

* Corresponding author

Email: [k.ellacott@exeter.ac.uk](mailto:k.ellacott@exeter.ac.uk)

# Supplementary Figures


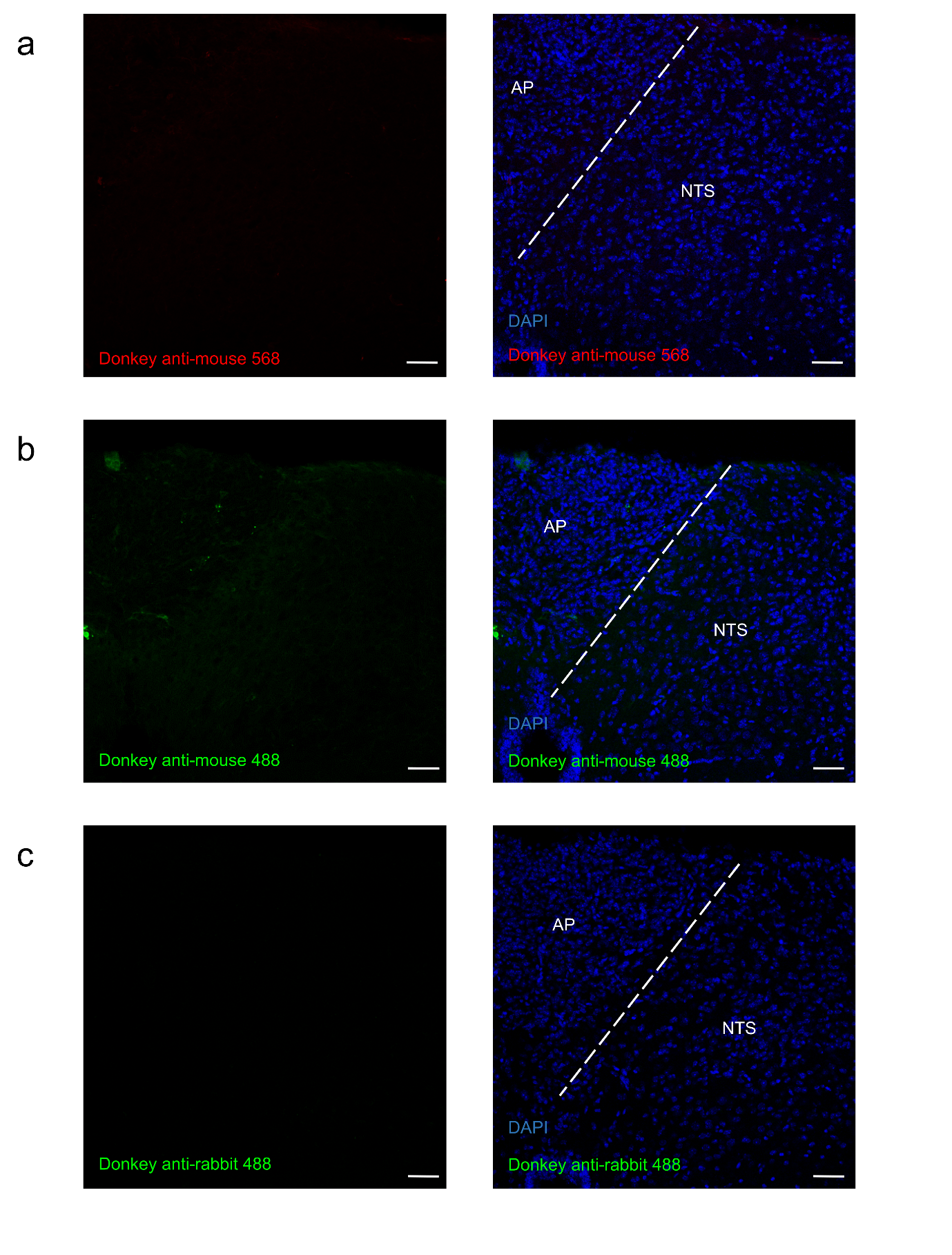


**Supplementary Figure 1 | Demonstration of specificity of antibody binding.** Representative images of sections stained in the absence of the primary antibody. **a,** donkey anti-mouse 568 **b,** donkey anti-mouse 488 **c**, donkey anti rabbit 488. Same section showing DAPI staining to right. Scale bar = 50 μm. AP = area postrema, NTS = nucleus of the solitary tract.


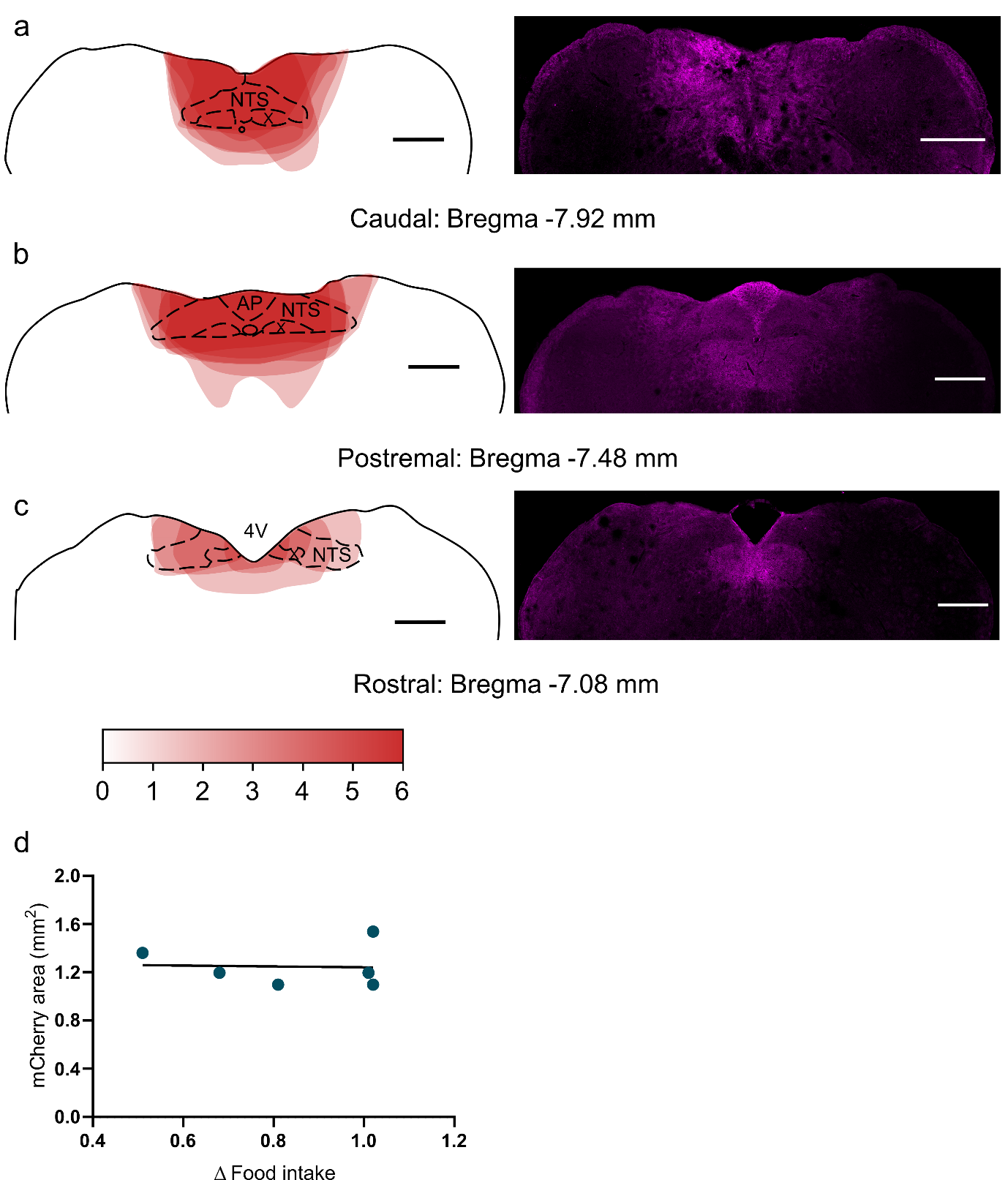


**Supplementary Figure 2 | AAV vector spread in individual DVC::GFAP^hM3Dq^ mice.** Visualization of mCherry reporter protein allowed mapping of the extent of transduction resulting from injection. **a-c,** Shown are the schematic diagrams of the patterns of mCherry expression and representative images from DVC:GFAP^hM3Dq^ mice (n = 6) at Bregma -7.92 mm **(a)**, Bregma -7.48 mm **(b)** and Bregma -7.08 mm **(c)**. Transduced regions are shown overlaid (darkest where all 6 mice overlap). **d,** Relationship between transduced area of postremal section (Bregma -7.48 mm) and the CNO-induced reduction of food intake (calculated as control food intake (saline) minus CNO food intake 4 hours post injection) (Linear regression slope = -0.04, not significantly different from zero p = 0.93). Scale bar = 500 μm. 4V = fourth ventricle, AP = area postrema, NTS = nucleus of the solitary tract, X = dorsal motor nucleus of the vagus.


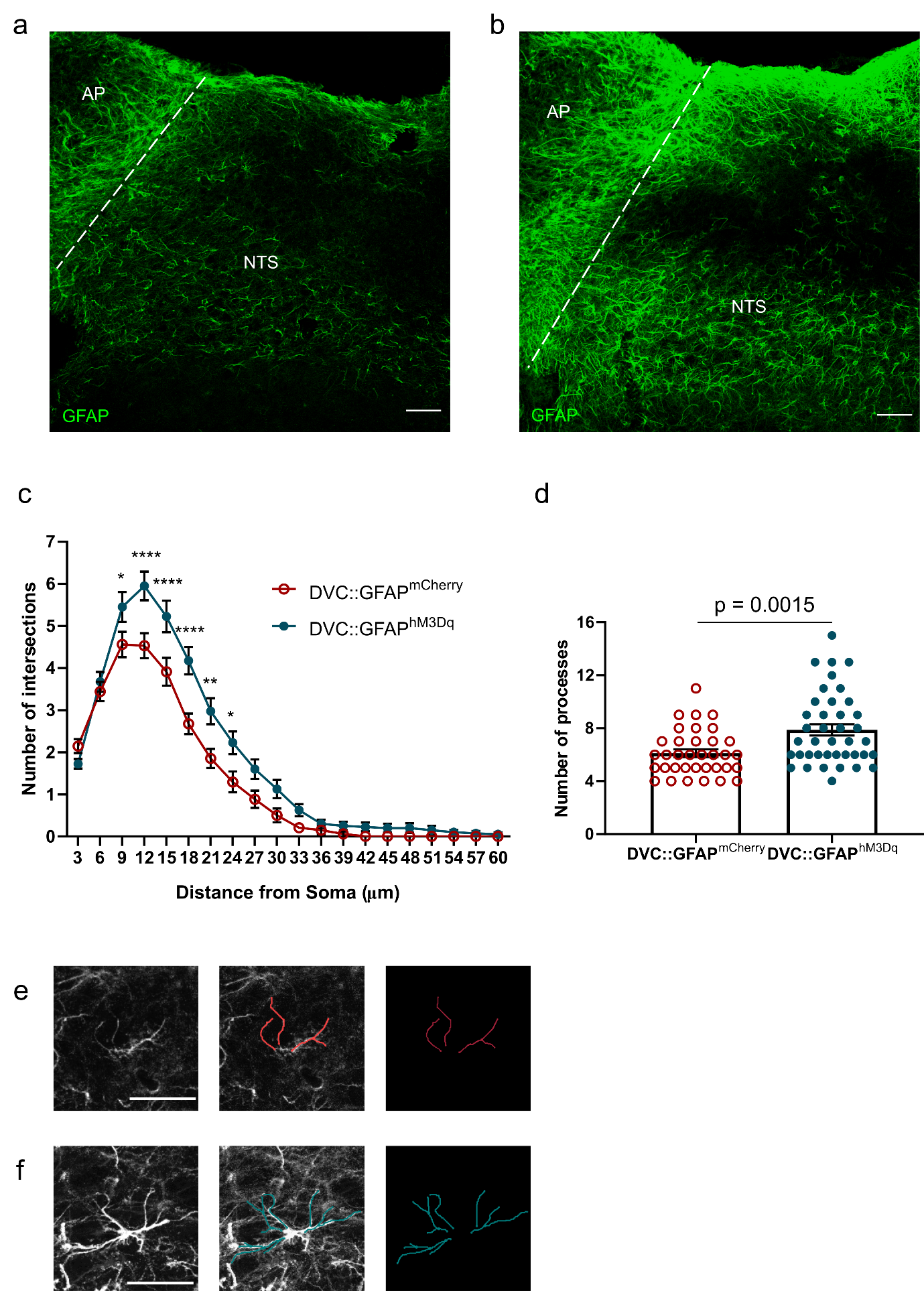


**Supplementary Figure 3 | Chemogenetic activation of DVC astrocytes increases morphological complexity.** DVC::GFAP^mCherry^ and DVC::GFAP^hM3Dq^ mice were injected with CNO (0.3 mg/kg) and perfused 2-3 hours later. **a,b,** Representative confocal image of GFAP immunostaining from a DVC::GFAP^mCherry^ **(a)** and a DVC::GFAP^hM3Dq^ **(b)** mouse, scale bar = 50 µm. **c,** Mean Sholl profile of postremal NTS astrocytes of DVC::GFAP^mCherry^ and DVC::GFAP^hM3Dq^ mice (n=35-40 cells from 4 mice/group, Two-way ANOVA, DREADD, p<0.0001, F_(1,1440)_=63.05; Distance from soma, p<0.0001, F_(19,1440)_=167.0; interaction, p=<0.0001, F_(19,1440)_=3.49; Sidak’s post-hoc test). **d,** Number of processes of individual postremal NTS astrocytes of DVC::GFAP^mCherry^ and DVC::GFAP^hM3Dq^ mice (6.14 ± 029 vs 7.88 ± 0.43, n=35-40 cells from 4 mice/group, p=0.0015, unpaired t-test). **e,f¸** Representative image and trace of a GFAP+ cell from a DVC::GFAP^mCherry^ **(e)** and a DVC::GFAP^hM3Dq^ **(f)** mouse, scale bar = 25 μm. * = p<0.05, ** = p<0.01, **** = p<0.0001. Data are expressed as mean ± standard error of the mean.


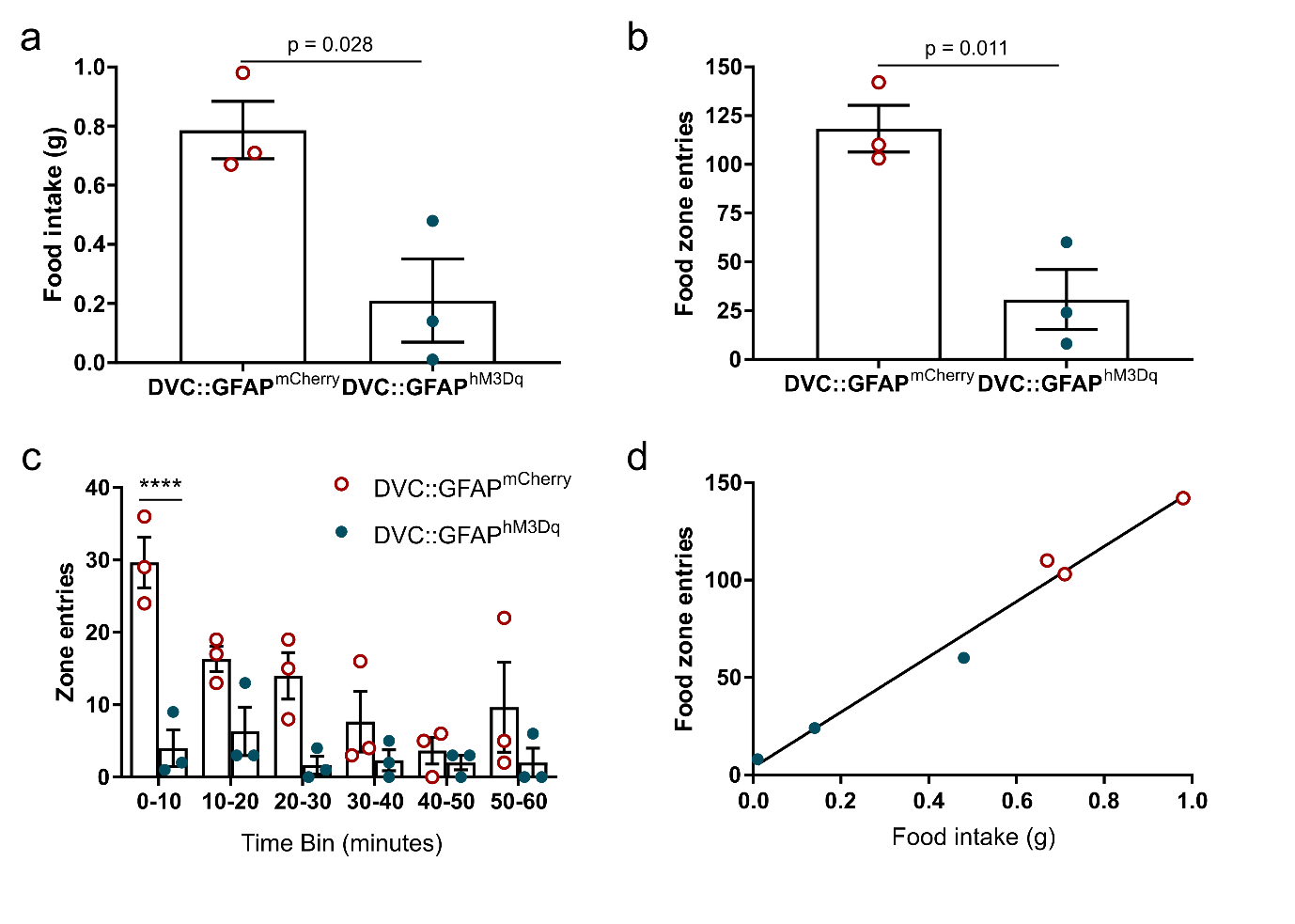


**Supplementary Figure 4 | Food seeking was lower in DVC::GFAP^hM3Dq^ than DVC::GFAP^mCherry^ mice following CNO injection.** DVC::GFAP^mCherry^ and DVC::GFAP^hM3Dq^ mice (n=3 mice/group) were injected with CNO at the beginning of the dark-phase and video monitored for 3 hours in their home cage with food pellets in the far corner from their nest. **a,** Food intake during the 3 hour monitoring period (0.79 ± 0.10 vs 0.21 ± 0.14 g, p=0.028, unpaired t-test). **b,** Total number of entries to the food containing zone of the cage made during the 3 hour monitoring period (118.3 ± 12 vs 30.67 ± 15.38 entries, p=0.011, unpaired t-test). **c,** Number of entries to the food containing zone of the cage in ten minute bins made by DVC::GFAP^mCherry^ (red) and DVC::GFAP^hM3Dq^ (blue) mice in the first hour of the monitoring period (Two-way ANOVA, DREADD, p=0.0052, F_(1,4)_=30.81; Time, p<0.0019, F_(5,20)_=5.74; interaction, p<0.013, F_(5,20)_=3.85, Sidak’s post-hoc test). **d,** relationship between total food intake and total entries to the food containing zone (linear regression slope = 141.8, significantly non-zero p = 0.0002). **** = p<0.0001. Data are expressed as mean ± standard error of the mean.


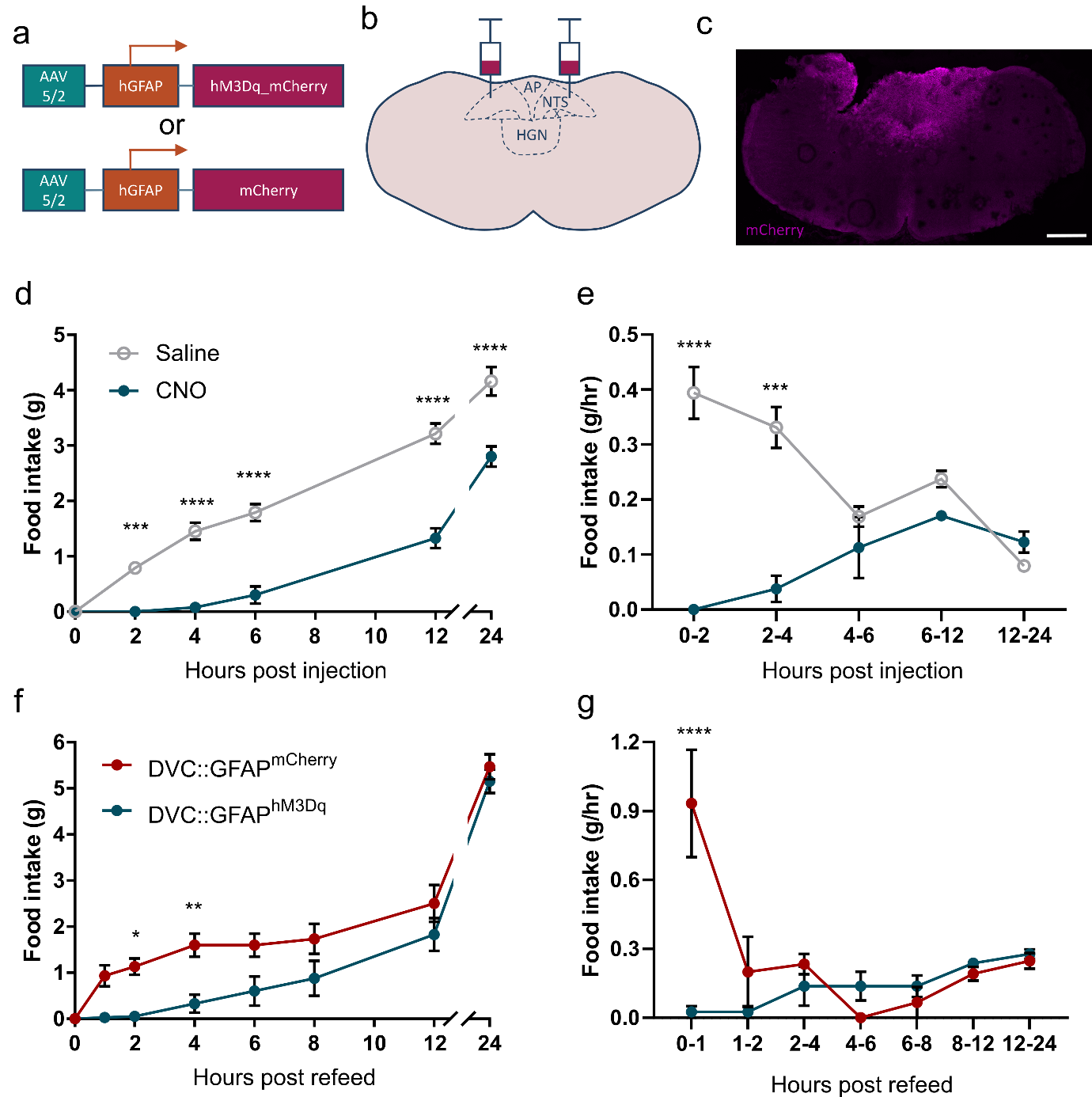


**Supplementary Figure 5 | Single AAV injection to DVC gives the same feeding suppressive effect. a,** AAV vectors containing either hM3Dq_mCherry or mCherry under the hGFAP promoter. **b,** Schematic of bilateral injection of the vector into the DVC. **c,** Representative image showing mCherry immunofluorescence in a DVC::GFAP^hM3Dq^ mouse, scale bar = 500 µm. **d, e,** DVC::GFAP^hM3Dq^ mice (previously transduced with a 180nl single vector injection bilaterally, n=4) were injected i.p. with saline or CNO (0.3 mg/kg) 30 minutes prior to the beginning of the dark-phase. **d,** Cumulative food intake (Two-way RM ANOVA, CNO, p<0.0001, F_(1,18)_=335.1; Time, p<0.0001, F_(5,18)_=112.0; interaction, p<0.0001, F_(5,18)_=18.64, Sidak’s post-hoc test). **e,** Rate of food intake (n=4 mice, Two-way RM ANOVA, CNO, p=0.0036, F_(1,3)_=69.45; Time, p=0.0019, F_(4,12)_=8.25; interaction, p<0.0001, F_(4,12)_=16.03, Sidak’s post-hoc test). **f, g,** DVC::GFAP^mCherry^ and DVC::GFAP^hM3Dq^ mice (n=3-4/group) were fasted for 12 hours during the dark phase then injected i.p. with CNO (0.3 mg/kg) 30 minutes prior to reintroduction of food at the onset of the light phase. **f,** Cumulative food intake (Two-way ANOVA, DREADD, p=0.038, F_(1,5)_=7.818; Time, p<0.0001, F_(7,35)_=168.0; interaction, p=0.026, F_(7,35)_=2.67, Sidak’s post-hoc test). **g,** Rate of food intake (Two-way ANOVA, DREADD, p=0.029, F_(1,5)_=9.29; Time, p=0.0001, F_(6,30)_=6.73; interaction, p<0.0001, F_(6,30)_=11.21, Sidak’s post-hoc test). * = p<0.05, ** = p<0.01, **** = p<0.0001. Data are expressed as mean ± standard error of the mean.
